# Metabolic syndrome and adverse clinical outcomes in patients with bipolar disorder

**DOI:** 10.1186/s12888-016-1143-8

**Published:** 2016-12-15

**Authors:** Ya-Mei Bai, Cheng-Ta Li, Shih-Jen Tsai, Pei-Chi Tu, Mu-Hong Chen, Tung-Ping Su

**Affiliations:** 1 Department of Psychiatry, Taipei Veterans General Hospital, Taipei, Taiwan; 2 Department of Psychiatry, College of Medicine, National Yang-Ming University, Taipei, Taiwan

**Keywords:** Metabolic syndrome, Bipolar disorder, Clinical outcome

## Abstract

**Background:**

Metabolic syndrome (MetS) is highly prevalent among patients with bipolar disorder. MetS may cause complications in the brain, but studies investigating MetS-associated clinical psychiatric outcomes remain scant.

**Methods:**

We enrolled clinically stable outpatients with bipolar disorder aged 18–65 years and performed anthropometric and fasting biochemical assessments to investigate MetS prevalence. We then performed clinical assessments by using the Young Mania Rating Scale for manic symptoms, the Montgomery–Åsberg Depression Rating Scale for depressive symptoms, the Positive and Negative Symptom Scale for psychotic symptoms, the Involuntary Movement Scale for tardive dyskinesia, the Barnes Akathisia Rating Scale for akathisia, the Udvalg for Kliniske Undersogelser for general side effects, the Schedule for Assessment of Insight for insight, the Global Assessment of Functioning scale for global functioning, and the Wisconsin Card Sorting Test (WCST) for cognitive executive function.

**Results:**

In total, 143 patients were enrolled and had a MetS prevalence of 29.4%. The patients treated with atypical antipsychotics plus mood stabilizers (36.3%) and atypical antipsychotics alone (36.0%) had a significantly higher prevalence of MetS than did those treated with mood stabilizers alone (10.5%; *p* = 0.012). According to multivariate regression analyses adjusted for age, sex, smoking status, bipolar disorder subtype (I or II), pharmacological treatment duration, and psychiatric medication, compared with patients without MetS, those with MetS had significantly more previous hospitalizations (*p* = 0.036), severer tardive dyskinesia (*p* = 0.030), poorer insight (*p* = 0.036), poorer global function (*p* = 0.046), and more impaired executive function (conceptual level response on the WCST; *p* = 0.042).

**Conclusions:**

Our results indicated that patients with comorbid bipolar disorder and MetS have more adverse clinical outcomes than those without, with more hospitalizations, severer tardive dyskinesia, poorer insight, poorer global function, and more impaired executive function. Monitoring MetS is crucial for assessing not only physical burden, but also psychiatric outcomes.

## Background

Metabolic syndrome (MetS) is highly prevalent (16.7%–67%) among patients with bipolar disorder in many countries [1–3]. Bipolar disorder and MetS have common risk factors, including endocrine disturbances, sympathetic nervous system dysregulation, and behavior patterns such as physical inactivity and overeating [4, 5]. In addition, commonly used pharmacological bipolar disorder treatments, such as mood stabilizers and antipsychotics, may intensify the medical burden by causing weight gain and metabolic disturbances, such as alterations in lipid and glucose metabolism [6, 7]. Comorbid MetS and bipolar disorder are associated with a high risk of cardiovascular disease [3, 5, 8], a two-fold increase in mortality [9], and a 10–20-year shortening of life expectancy [10]. Studies have shown that obesity and metabolic diseases are associated with poor clinical outcome in bipolar disorder. Fagiolini et al. reported that 35.4% of 175 patients with bipolar disorder were obese and that these patients had more previous depressive and manic episodes as well as exhibited a significantly shorter time to recurrence during the maintenance phase than nonobese patients did [11]. Similarly, Calkin et al. reported that 39.1% of 276 patients with bipolar disorder were obese and that these patients had a longer illness duration, poorer global function, more disability, and poorer response to lithium than nonobese patients did [12]. In addition, obesity in bipolar disorder is associated with illness severity, particularly in relation to depression [13–15], unfavorable course of illness [15], and poor cognitive function [16–19]. Compared with euglycemic patients, those with bipolar disorder and type 2 diabetes or insulin resistance have significantly higher risk of a chronic course, rapid cycling, and bipolar disorder refractory to lithium treatment [20–22].

Nevertheless, studies investigating MetS-associated clinical psychiatric outcomes remain scant. MetS is a multidimensional entity that includes visceral obesity, dyslipidemia, hyperglycemia, and hypertension. MetS may cause complications in the brain: In an animal study, chronic hyperglycemia and insulin resistance were reported as risk factors for neuronal death through induction of a state of oxidative stress and inflammatory response, thus affecting cognitive processes [23]. Patients with MetS showed a significant reduction in mean cortical thickness and volume in both hemispheres compared with controls, suggesting an initial neurodegenerative process and cognitive deterioration with MetS, even at a preclinical stage [24]. Furthermore, four studies of topiramate as an add-on therapy in patients with bipolar disorder showed that weight reduction is associated with a significant reduction in both depressive and manic symptoms [25–28]. These interventions are potentially efficacious in ameliorating disturbed biological pathways, particularly those mediating inflammation and oxidative stress, and reducing the rate of neuroprogressive disturbances in bipolar disorders [29]. In this study, we investigated the association between MetS and clinical outcomes of patients with bipolar disorder. We hypothesized that patients with comorbid bipolar disorder and MetS have more adverse clinical outcomes than those without MetS do.

## Methods

This study was conducted in a psychiatric outpatient clinic of a university medical center hospital. The patient inclusion criteria were as follows: outpatients with bipolar disorder (DSM-IV), and those aged 18–65 years with a Clinical Global Impression-Severity rating for bipolar disorder of ≤3. The exclusion criteria were any DSM-IV diagnosis of a lifetime history of schizophrenia, mental retardation, or organic mental disorder, and those currently pregnant or breastfeeding. All patients provided written informed consent before inclusion. The study was approved by the Institutional Review Board of Taipei Veterans General Hospital and conducted in accordance with the Declaration of Helsinki.

The medical and psychiatric histories of the patients were reviewed. Anthropometric and fasting biochemical assessments were performed to investigate MetS prevalence according to the 2005 International Diabetes Federation Asia criteria; a waist circumference of >90 cm in men or >80 cm in women was the essential criterion of central obesity in addition to any two of the following criteria: (a) fasting serum triglyceride levels of ≥150 mg/dL, (b) fasting high-density lipoprotein cholesterol levels of <40 mg/dL in men or <50 mg/dL in women, (c) blood pressure of ≥130/85 mmHg, or (d) a fasting glucose level of ≥100 mg/dL. Patients receiving medication for hypertension, diabetes, or hyperlipidemia were considered to fulfill the MetS components criteria. We used the following scales for clinical assessment: the Young Mania Rating Scale (YMRS) for manic symptoms, the Montgomery–Åsberg Depression Rating Scale (MADRS) for depressive symptoms, the Positive and Negative Symptom Scale (PANSS) for psychotic symptoms, the Simpson–Angus Scale (SAS) for extrapyramidal side effects [30], the Abnormal Involuntary Movement Scale (AIMS) for tardive dyskinesia [31], the Barnes Akathisia Rating Scale (BARS) for akathisia [32], the Udvalg for Kliniske Undersogelser for general side effects [33], the Schedule for Assessment of Insight (SAI) for insight [34], and the Global Assessment of Functioning (GAF) scale for functioning. Furthermore, cognitive executive function was assessed using the Wisconsin Card Sorting Test (WCST). All clinical assessments were conducted by Dr. Bai (first author).

Patient characteristics were evaluated through descriptive analyses. Patients with and without MetS were compared using a chi-square test for categorical data and the Student *t* test for continuous data. The Fisher exact test was used if fewer than five data points were expected to be greater than 20% of the cells in the contingency table. All tests were based on their two-tailed alternatives. The clinical outcomes of the patients with and without MetS were further compared using multivariate regression analyses adjusted for age, sex, smoking status, bipolar disorder subtype (I or II), pharmacological treatment duration, and psychiatric medication (monotherapy or combination therapy). The level of significance was set at a *p* value of 0.05. All statistical analyses were performed using SPSS 11.5 (SPSS Inc., Chicago, IL, USA).

## Results

In total, we enrolled 143 outpatients with bipolar disorder (66.4% women) with an average age of 44.8 ± 12.0 years. The prevalence of MetS was 29.4%. Age, sex, education, pharmacological treatment duration for bipolar disorder, suicide history, bipolar subtype (I or II), as well as severity of manic (YMRS), depressive (MADRS), and psychotic (PANSS) symptoms did not differ significantly between the patients with and without MetS. The patients treated with atypical antipsychotics plus mood stabilizers (36.3%) and atypical antipsychotics alone (36.0%) had a significantly higher MetS prevalence than did those treated with mood stabilizers alone (10.5%; *p* = 0.012). Univariate analysis showed that compared with the patients without MetS, those with MetS had significantly more previous hospitalizations (*p* = 0.024), more first mood episodes with mania (*p* = 0.033), more extrapyramidal side effects (SAS; *p* = 0.003), higher incidence of akathisia (BARS; *p* = 0.034), higher incidence of involuntary movement disorder (AIMS; *p* = 0.043), poorer global functioning (GAF; *p* = 0.035), poorer insight (SAI; *p* = 0.027), and more impaired executive function (conceptual level response on the WCST; *p* = 0.007). The patients with MetS also had significantly more medical comorbidities than those without MetS did (71.4% vs. 32.3%, *p* < 0.0001), including hypertension (54.8% vs. 13.9%, *p* < 0.0001), hyperlipidemia (26.2% vs. 5.9%, *p* = 0.001), and diabetes mellitus (23.8% vs. 4%, *p* = 0.001).

**Fig. 1:**
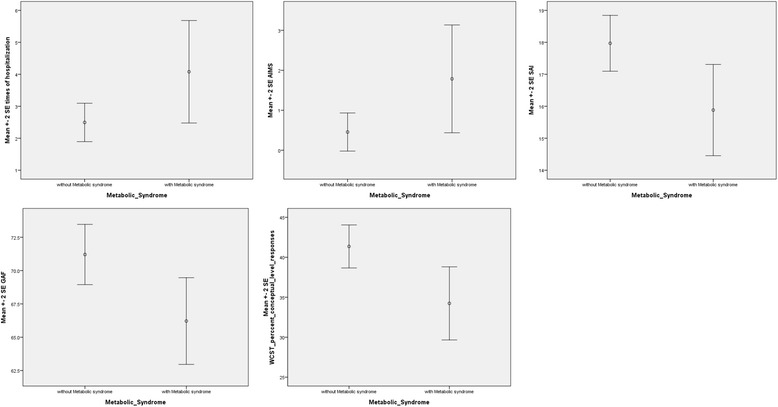
Comparison of clinical outcomes between patients with and without metabolic syndrome using multivariate regression analyses with adjustments of age, gender, smoking, subtype of bipolar disorder, pharmacological treatment duration, and psychiatric medication group

Because smoking status [35, 36], bipolar disorder subtype [37], illness duration [38, 39], and psychiatric medications [40, 41] may influence clinical outcomes, we used multivariate regression analyses adjusted for age, sex, smoking status, bipolar disorder subtype (I or II), pharmacological treatment duration, and psychiatric medication (mood stabilizers alone, atypical antipsychotics alone, or atypical antipsychotics plus mood stabilizers). We observed that compared with the patients without MetS, those with MetS had significantly more previous hospitalizations (*p* = 0.036), severer tardive dyskinesia (AIMS; *p* = 0.030), poorer insight (SAI; *p* = 0.036), poorer global function (GAF; *p* = 0.046), and more impaired executive function (conceptual level response on the WCST; *p* = 0.042; Fig. 1; Tables 1 and 2).

**Table 1:** Comparison of characteristics between bipolar patients with and without metabolic syndrome

	Patients with metabolic syndrome (*n* = 42)	Patients without metabolic syndrome (*n* = 101)	df, *p*-value
Age (year)	47.5 ± 11.2	43.7 ± 12.2	n.s.
Gender (F, %)	67.9%	69.8%	n.s.
Education (more than 12 years %)	33.3%	33.7%	n.s.
Occupation (regular work %)	28.6%	33.7%	n.s.
Age at onset of bipolar disorder	31.5 ± 12.7	28.4 ± 11.7	n.s.
Treatment Duration of bipolar disorder	13.9 ± 11.2	13.1 ± 10.7	n.s.
Smoking (%)	31%	23.8%	n.s.
Times of hospitalization	4.1 ± 4.9	2.5 ± 2.9	df = 47.7,*P* = 0.024
History of suicide attempt (%)	42.9%	47.5%	n.s.
Subtype of bipolar disorder (bipolar I %)	69%	59.4%	n.s.
Pharmacological treatment groups	52.4%	43.6%	n.s.
[mood stabilizers only]	9.5%	33.7%	df = 2,*P* = 0.012
[atypical antipsychotics only]	21.4%	15.8%	
[atypical antipsychotics plus mood stabilizers]	69%	50.5%	
First mood episode with mania/hypomania (%)	52.5%	32.0%	df = 1,*P* = 0.033
Young Mania Rating Scale (YMRS)	5.3 ± 5.0	4.7 ± 4.9	n.s.
Montgomery Åsberg Depression Rating Scale (MADRS)	8.2 ± 7.4	9.2 ± 9.1	n.s.
Positive and Negative Symptom Scale (PANSS)	41.6 ± 12.6	39.4 ± 11.6	n.s.
Simpson-Angus Scale (SAS)	3.6 ± 4.7	2.1 ± 4.0	df = 67.6,*P* = 0.046
Abnormal Involuntary Movement Scale (AIMS)	1.8 ± 4.4	0.5 ± 2.4	df = 51.5,*P* = 0.021
Barnes Akathesia scale (BANS)	0.1 ± 0.6	0.2 ± 0.9	n.s.
Udvalg for Kliniske Undersogelser (UKU)	5.7 ± 3.3	4.7 ± 4.0	n.s.
Schedule for Assessment of Insight (SAI)	16.2 ± 4.9	18.1 ± 4.4	df = 140,*P* = 0.027
Global Assessment of Functioning scale (GAF)	67.1 ± 11.7	71.5 ± 11.0	df = 141,*P* = 0.035
Level of lithium (*n* = 33)	0.51 ± 0.21	0.74 ± 0.62	n.s.
Level of valproic acid (*n* = 52)	55.0 ± 35.3	40.8 ± 28.5	n.s.
Level of carbamazapine (*n* = 4)	--	6.2 ± 1.7	--
Wisconsin Card Sorting Test (WCST) Conceptual level response %	34.2 ± 12.5	41.4 ± 11.7	df = 104,*P* = 0.007
Body Mass Index (BMI)	30.2 ± 4.2	24.8 ± 4.8	df = 141,*P* < 0.0001
waist circumference > 90 cm in males or > 80 cm in females	100%	42.6%	df = 1,*P* < 0.0001
fasting serum triglyceride levels≧150 mg/dL	69%	14.9%	df = 1,*P* < 0.0001
fasting high density lipoprotein (HDL) cholesterol < 40 mg/dL in men or <50 mg/dL in women	73.8%	13.9%	df = 1,*P* < 0.0001
blood pressure≧130/85 mmHg	78.6%	28.7%	df = 1,*P* < 0.0001
fasting glucose≧100 mg/dL	47.6%	10.9%	df = 1,*P* < 0.0001
Waist circumference (cm)	97.1 ± 9.7	81.3 ± 11.2	df = 100,*P* < 0.0001
Systolic blood pressure (mmHg)	131.5 ± 17.1	120.6 ± 16.9	df = 141,*P* = 0.001
Diastolic blood pressure (mm Hg)	82.1 ± 10.5	75.9 ± 11.4	df = 141,*P* = 0.003
Triglyceride (microg/ dL)	198.4 ± 122.1	101.4 ± 69.4	df = 52.4,*P* < 0.0001
High density lipoprotein (HDL) (microg/ dL)	41.6 ± 9.3	60.9 ± 15.6	df = 123.6,*P* < 0.0001
Glucose (microg/ dL)	101.8 ± 37.7	87.7 ± 16.9	df = 46.7,*P* = 0.026
Insulin (pg/ml)	13.7 ± 10.7	8.0 ± 13.6	df = 133,*P* = 0.019

**Table 2:** Multivariate regression analyses for clinical outcome and pro-inflammatory cytokines

	Previous hospitalizations (times)		Dyskinesia (AIMS)		Insight(SAI)		Global function (GAF)		Executive function(WCST)	
	Standardized coefficient (ß) (95% C.I.)	*P* value	Standardized coefficient (ß) (95% C.I.)	*P* value	Standardized coefficient (ß) (95% C.I.)	*P* value	Standardized coefficient (ß) (95% C.I.)	*P* value	Standardized coefficient (ß) (95% C.I.)	*P* value
Age	−0.18	0.848	***0.190***	0.020**	−0.063	0.479	−0.054	0.526	***−0.392***	P < 0.001**
	(−0.062,0.051)		***(0.03,0.094)***		(−0.092,0.043)		(−0.208,0.107)		***(−0.628,-0.205)***	
Gender										
Male	--	--	--	--			--	--	--	--
Female	***0.205***	0.021**	***0.203***	0.020**	***0.190***	0.026**	−0.144	0.079	−0.044	0.653
	***(0.238,2.918)***		***(0.215,2.419)***		***(0.224,3.464)***		(−7.21,0.397)		(−5.945,3.741)	
Smoking	0.072	0.415	0.141	0.105	0.049	0.565	***−0.238***	0.004**	−0.137	0.154
	(−0.870,2.095)		(−0.209,2.169)		(−1.240,2.262)		***(−10.170,-1.962)***		(−0.9007,1.445)	
Bipolar disorder subtype										
Type I	--	--	--	--			--	--	--	--
Type II	***−0.197***	0.028**	−0.408	0.684	***0.294***	0.001**	−0.009	0.911	−0.041	0.670
	***(−2.775,-0.162)***		(−1.305,-0.858)		***(1.194,4.385)***		(−3.944,3.522)		(−5.739,3.704)	
Pharmacological treatment duration (years)	***0.259***	0.005**	1.165	0.246	***0.258***	0.004**	0.154	0.071	0.083	0.401
	***(0.028,0.153)***		(−0.021,0.080)		***(0.035,0.183)***		(−0.014,0.332)		(−0.127,0.315)	
Psychiatric medication group										
Mood stabilizers only	--	--	--	--			--	--	--	--
Atypical antipsychotics only	0.185	0.071	0.094	0.355	0.054	0.586	***−0.321***	0.001**	***−0.219***	0.039**
	(−0.163,3.848)		(−0.852,2.357)		(−1.707,3.006)		***(−15.003,-3.928)***		***(−15.303,-0.396)***	
Atypical antipsychotics plus mood stabilizers	0.089	0.383	0.049	0.630	0.139	0.164	***−0.312***	0.001**	***−0.218***	0.042**
	(−0.829,2.143)		(−0.937,1.543)		(−0.534,3.124)		***(−11.365,-2.805)***		***(−10.721,-0.196)***	
Metabolic syndrome (+)	***0.19***	0.036**	***2.190***	0.030**	***−0.179***	0.036**	***−0.164***	0.046**	***−0.324***	0.042**
	***(0.097,2.918)***		***(0.121,2.387)***		***(−3.447,-0.118)***		***(−7.891,-0.071)***		***(−9.969,-0.178)***	

** p<0.05

The bold-italic data emphasized the statistical significance

## Discussion

Our study showed that the prevalence of MetS was 29.4% among patients with bipolar disorder, comparable to the previously reported 33.9% in Taiwan [42] and 16.7%–67% in other countries [1]. The frequent cooccurrence of bipolar disorder and MetS may be characterized by the common genetic links, interconnected pathophysiologies, and interacting biological networks with structural and functional abnormalities in multiple cortical and subcortical brain regions subservient to cognitive and affective processing [1, 4, 5, 29]. Furthermore, many commonly used pharmacological treatments for bipolar disorder, antipsychotics, and mood stabilizers increase both weight and MetS risk [1, 3, 43, 44]. In this study, we demonstrated that patients treated with atypical antipsychotics plus mood stabilizers and atypical antipsychotics alone had a significantly higher MetS prevalence than those treated with mood stabilizers alone did. Our results were consistent with previous study results, in which cotreatment with mood stabilizers plus antipsychotics increased MetS risk [44, 45]. A comparative analysis of 32 double-blind, randomized, placebo-controlled trials showed that antipsychotics caused more weight gain than did mood stabilizers in youths [46]. Currently, many atypical antipsychotics are recommended as first-line monotherapy or in combination with mood stabilizers to improve the efficacy of bipolar disorder treatment [40, 41, 47–49]. Our results suggest that efficacious advantages of antipsychotics in bipolar disorder treatment should be balanced with their greater MetS risk.

To investigate the role of MetS in the clinical outcomes, we used multivariate regression analyses adjusted for age, sex, smoking status, bipolar disorder subtype (I or II), pharmacological treatment duration, and psychiatric medication (mood stabilizers alone, atypical antipsychotics alone, or atypical antipsychotics plus mood stabilizers) because smoking status [35, 36], bipolar disorder subtype [37], illness duration [38, 39], and psychiatric medications [40, 41] potentially influence the clinical outcomes. In addition, we observed that MetS was associated with more previous hospitalizations, severer tardive dyskinesia, poorer insight, poorer global functioning, and more impaired cognitive executive function. The current results supported our hypothesis that patients with comorbid bipolar disorder and MetS associate with the severity of neurodegenerative processes and cognitive impairment than those without [23, 50]. MetS can elicit inflammatory response (increase in the number of reactive astrocytes as well as the levels of interleukin-1-beta and tumor necrosis factor-alpha) and oxidative stress (reactive oxygen species and lipid peroxidation), causing a reduction in the number of neurons in the temporal cortex and hippocampus [23, 51]. Hyperglycemia can directly affect neuron myelin or axons and the structure and function of endoneurial microvessels, which can then induce fiber changes by altering the blood–nerve barrier and inducing hypoxia or ischemia or through unknown mechanisms [52]. The positive association between MetS and cognitive dysfunction was documented with volume losses in the hippocampus and frontal lobes [53]. Significant correlations between brain microstructural white matter alterations and cognitive impairment have also been noted in patients with MetS, particularly in the frontal lobe [54]. MetS negatively affects cognitive performance and brain structure. Potential explanatory models include impaired vascular reactivity, neuroinflammation, oxidative stress, and abnormal brain lipid metabolism [53]. Patients with MetS may also experience a substantial negative economic and health impact, considerably reducing their quality of life and social function [11, 12, 55–57].

Notably, in multivariate regression analyses, our patients with comorbid bipolar disorder and MetS had severer tardive dyskinesia than did those without. Studies have suggested that patients with affective disorder treated with antipsychotics have a greater risk of tardive dyskinesia [58]. Nevertheless, we adjusted for the pharmacological treatment duration and psychiatric medication (mood stabilizers alone, atypical antipsychotics alone, or atypical antipsychotics plus mood stabilizers) in the multivariate regression analyses. The association between MetS and tardive dyskinesia cannot be entirely explained by the pharmacological treatment. The pathophysiology of tardive dyskinesia remains to be completely understood. In addition to the leading hypothesis of dopamine receptor hypersensitivity with antipsychotic treatment, a crucial pathophysiological theory of tardive dyskinesia involves neurotoxicity [59]. MetS can elicit inflammatory response and oxidative stress, reducing the number of neurons [23, 51]; animal studies have shown that tardive dyskinesia is associated with inflammation and apoptosis [60, 61]. Furthermore, the potent antioxidants vitamins E and B6 as well as piracetam have been shown to alleviate the severity of tardive dyskinesia in randomized, double-blind, placebo-controlled studies [62, 63]. In addition, the severity of tardive dyskinesia is positively associated with that of cognitive dysfunction [64–66], significantly reduced gray matter, and widespread abnormality of white matter over corticobasal ganglion circuits, which involves emotional and behavioral regulation as well as executive function [67, 68]. Taken together, these studies further support our results that patients with MetS have severer tardive dyskinesia and are characterized by severer neurodegeneration and poorer clinical outcomes.

### Strengths and limitations

Our study investigated the association between MetS and clinical outcomes in patients with bipolar disorder. The current results indicate that monitoring MetS is important for both physical and psychiatric outcomes in patients with bipolar disorder. Although the exact causes of MetS in bipolar disorder vary across patients, the etiological cascade—including biological, psychological, and sociodemographic variables— ultimately affects the clinical outcomes of the patients, whether directly or indirectly. The results also highlight the need for collaborative care among psychiatric and general medical providers to concomitantly address the psychiatric and other medical needs of patients with bipolar disorder. However, our study has some limitations, mainly because of its cross-sectional design. First, whether the association between MetS and adverse clinical outcomes of bipolar disorder is causal remains to be determined. The presence of MetS may lead to more severe neurodegeneration process and cause adverse clinical outcomes; by contrast, patients with poorer clinical outcomes may develop MetS because they receive more complex combination therapy. Nevertheless, even more, probable is the pathophysiology change of MetS and adverse clinical outcomes occur simultaneously and interact intermingle with each other. Long-term studies are needed to elucidate whether the processes of MetS and clinical outcomes are distinguishable. Second, even though the current psychiatric medication groups were controlled for in the multivariate regression analyses, the impacts of previous psychiatric medications were difficult to investigate. The average treatment duration was 13 years among our patients, and their previous medications involved varying combinations and treatment durations. Thus, clarifying the role of a specific medication in the development of MetS as well as their influence on clinical outcomes or cognitive function was difficult. Prospective studies examining patients with the newly diagnosed bipolar disorder are required. Third, our study participants were clinically stable outpatients; hence, a generalization of the results to the entire population of patients with bipolar disorder may be limited.

## Conclusion

The prevalence of MetS in the 143 clinically stable outpatients with bipolar disorder was 29.4%. The patients treated with atypical antipsychotics plus mood stabilizers (36.3%) and atypical antipsychotics alone (36.0%) had a significantly higher prevalence of MetS than did those treated with mood stabilizers alone (10.5%). The efficacy advantages of antipsychotics in bipolar disorder should be balanced with its greater risk of MetS. The patients with comorbid bipolar disorder and MetS have adverse clinical outcomes than those without, with more hospitalizations, more tardive dyskinesia, poorer insight, poorer global function, and more impaired executive function. The current results supported our hypothesis that patients with comorbid bipolar disorder and MetS associated with the severity of neurodegenerative processes than those without. Monitoring of metabolic syndrome is not only important for physical burden, as considerably as for psychiatric outcomes.
